# Palaeo-tsunami inundation distances deduced from roundness of gravel particles in tsunami deposits

**DOI:** 10.1038/s41598-019-46584-z

**Published:** 2019-07-16

**Authors:** Daisuke Ishimura, Keitaro Yamada

**Affiliations:** 1 Department of Geography, Tokyo Metropolitan University 1-1 Minami-Osawa, Hachioji City, Tokyo 192-0397 Japan; 2 Research Centre for Palaeoclimatology, Ritsumeikan University 122-12-1 Torihama, Wakasa-cho, Mikatakaminaka-gun, Fukui Prefecture 919-1331 Japan

**Keywords:** Natural hazards, Geomorphology, Sedimentology

## Abstract

Information on palaeo-tsunami magnitude is scientifically and socially essential to mitigate tsunami risk. However, estimating palaeo-tsunami parameters (e.g., inundation distance) from sediments is not simple because tsunami deposits reflect complex transport processes. Here, we show a new approach to estimate tsunami inundation distance based on the mixture ratio of gravels from several sources in tsunami deposits. We measured the roundness of source gravels in modern beach and fluvial deposits in a coastal valley in Japan through image analysis and then calculated the mixture ratio of both sediment types in tsunami deposits. Normalising the mixture ratios by inundation distances revealed an abrupt change in the mixture ratio at a constant percentile, regardless of tsunami magnitude. This relation allowed estimation of the inundation distance of palaeo-tsunamis during the last 4000 years.

## Introduction

Tsunamis are one of the most devastating natural hazards on Earth, triggered by earthquakes, submarine or onshore landslides, volcanic eruptions and meteorites. Thus, knowledge of palaeo-tsunami parameters (e.g., inundation distance, run-up height, and flow speed) is critical for disaster prevention and city planning along coastal regions. However, not only observed and documented historical records but also geological records must be used to understand low-frequency large tsunamis because recurrence intervals of tsunamigenic great earthquakes in subduction zones range from 10^2^–10^3^ years^1–5^. In particular, the AD 2004 Sumatra-Andaman earthquake (Indian Ocean tsunami) and the AD 2011 Tohoku-oki earthquake (Tohoku-oki tsunami) provided data as modern analogues of tsunamis triggered by earthquakes, and palaeo-tsunami research progressed and raised new problems^6,7^. For palaeo-tsunami parameters, inundation distance and area and run-up height can be estimated by palaeo-tsunami deposit distribution with palaeo-coastline reconstruction^8–10^, and flow speed can be reconstructed using numerical modelling^11–13^.

In this study, we estimated the palaeo-tsunami inundation distance (ID), which is an important parameter for quantifying the magnitude of a tsunami, using typical tsunami deposits from a small coastal valley in Japan.

We used Wadell’s definition of roundness^14^ (hereafter simply “roundness”)—a widely used shape-descriptive parameter—to characterise clasts within tsunami deposits by means of image analysis. This roundness, which is quite different from other shape parameters such as aspect ratio and circularity^15^, is calculated using radii of curvature for each corner and the maximum inscribed circle. Image analysis allows roundness data to be obtained on the order of 10^4^, two orders of magnitude greater than that obtained by conventional methods^16,17^, such as hand measurement and visual classification^18^. This result is a great improvement because it is difficult to perform detailed statistical analysis due to lack of roundness data from conventional methods. The large datasets of unique and sensitive roundness enable distinguishing beach and fluvial grains statistically. Assuming that the roundness distribution of tsunami deposits reflects the roundness distributions in source materials (beach and fluvial deposits), we calculated the mixture ratio of the source materials of the tsunami deposits. Consequently, we determined the relationship between the mixture ratio of historical tsunami deposits and normalised distances from the coastline by their observed IDs. Furthermore, we discovered that the mixture ratios show abrupt changes at a constant percentile in normalised distance, regardless of tsunami magnitude. Using this finding, we estimate unknown IDs of palaeo-tsunamis.

## Setting

**Figure 1:**
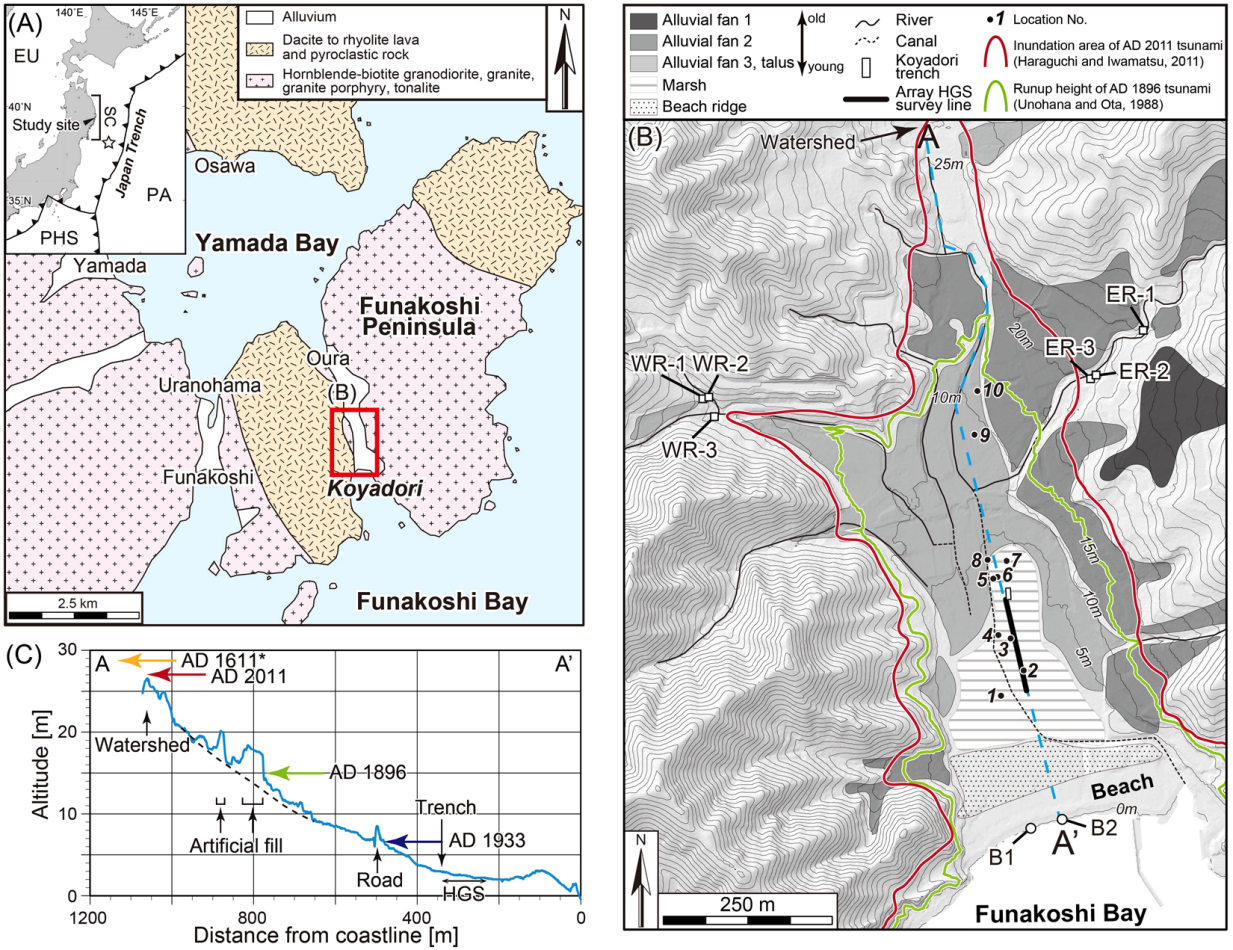
Location, geological setting, and topography of the study site. (**A**) Geology of the area around Koyadori, Iwate Prefecture, Japan^34^. EU: Eurasian Plate. PA: Pacific Plate. PHS: Philippine Sea Plate. SC: Sanriku Coast. The star indicates the epicentre of the 2011 Tohoku-oki earthquake. (**B**) Geomorphological map^21^. The contour map is based on the 1-m mesh digital elevation model supplied by Iwate Prefecture. The contour interval is 5 m. (**C**) A–A’ profile based on the 1-m mesh digital elevation model. The dates and arrows denote historical tsunami event ages and maximum run-up heights. *According to local tradition, the AD 1611 tsunami passed the watershed (at approximately 30 m above sea level) between Koyadori and Oura^35^. (**A**,**B**) are shared under a CCBY 4.0 open access licence (https://creativecommons.org/licenses/by/4.0/) and changes were made to the original figure.

The study site, Koyadori, where the AD 2011 Tohoku-oki tsunami ran up to approximately 30 m above sea level^19,20^, is a V-shaped, narrow coastal valley on the Sanriku Coast (Fig. 1B). Volcanic and pyroclastic rocks crop out on the west side of the Koyadori valley, and plutonic rocks crop out on the opposite side (Fig. 1A). However, the beach sediments are mainly composed of very coarse sand to pebbly gravel originating from volcanic and pyroclastic rocks^21^ (see Supplementary Fig. S1) because plutonic rocks are easily reduced to small sand size clasts and transported to offshore areas. In contrast, fluvial sediments comprise talus and alluvial fans (Fig. 1B) and are distributed narrowly on the riverbed and widely on the surfaces of the talus and alluvial fans.

As for palaeo-topography, the present topographic setting (depression between beach ridge and talus/alluvial fan) was already established in ca. 6000 cal yr BP based on a drilling survey and interbedded tephra age^21^. No marine deposits were interbedded within drilled core sediments in the Koyadori valley after 6000 cal yr BP. Additionally, the long-term subsidence rate using Holocene intertidal deposits in Miyako, approximately 20 km north of Koyadori, is less than 1.1–1.9 mm/yr^22^. These results indicate that the palaeo-coastline since ca. 6000 cal yr BP was in the same position as at present or more seaward.

The Tohoku-oki tsunami struck the Pacific coast of northeast Japan. Particularly high run-up heights were recorded along the Sanriku Coast^23^ (Fig. 1A). Historical records of tsunamis in this region are available for the last 400 years, and the run-up heights of historical tsunamis at Koyadori were estimated and measured (Table 1). Eleven modern and palaeo-tsunami deposits (E1 to E11, from youngest to oldest) that formed during the last 4000 years were previously identified at the Koyadori trench (12 m long, 3 m wide, and 2 m deep) excavated in a small lowland area of the valley (Fig. 1B) and dated by radiocarbon dating and tephrochronology^21,24^ (see Supplementary Table S1). The historical events corresponding to the E1, E2, and E3 tsunami deposits represent the AD2011 Tohoku-oki tsunami, the AD1896 Meiji Sanriku tsunami, and the AD1611 Keicho tsunami, respectively^21,24^.

**Table 1:** Information on historical tsunamis at Koyadori.

Correlated tsunami deposits at Koyadori trench	Tsunami event	Age [AD]	Earthquake magnitude	Earthquake type	Run-up height [m]	Inundation distance [m]	Reference No.
E1	Tohoku-oki	2011	9.0	Megathrust earthqauke	26.0–29.4	1100	^19, 20^
	Showa Sanriku	1933	8.1^36^	Outer-rise earthquake^37^	6.6	500	^25^
E2	Meiji Sanriku	1896	8.2^36^	Tsunami earthquake or slow earthquake^37^	15.0	800	^26^
E3	Keicho	1611	8.1^36^	Tsunami earthquake^37^	20–25*	1250	^27^

*In local tradition, the AD 1611 tsunami passed the watershed (about 30 m in altitude) between Koyadori and Oura^35^. Thus, we consider this value to be the minimum run-up height.

### Roundness measurements and mixture ratios

**Figure 2:**
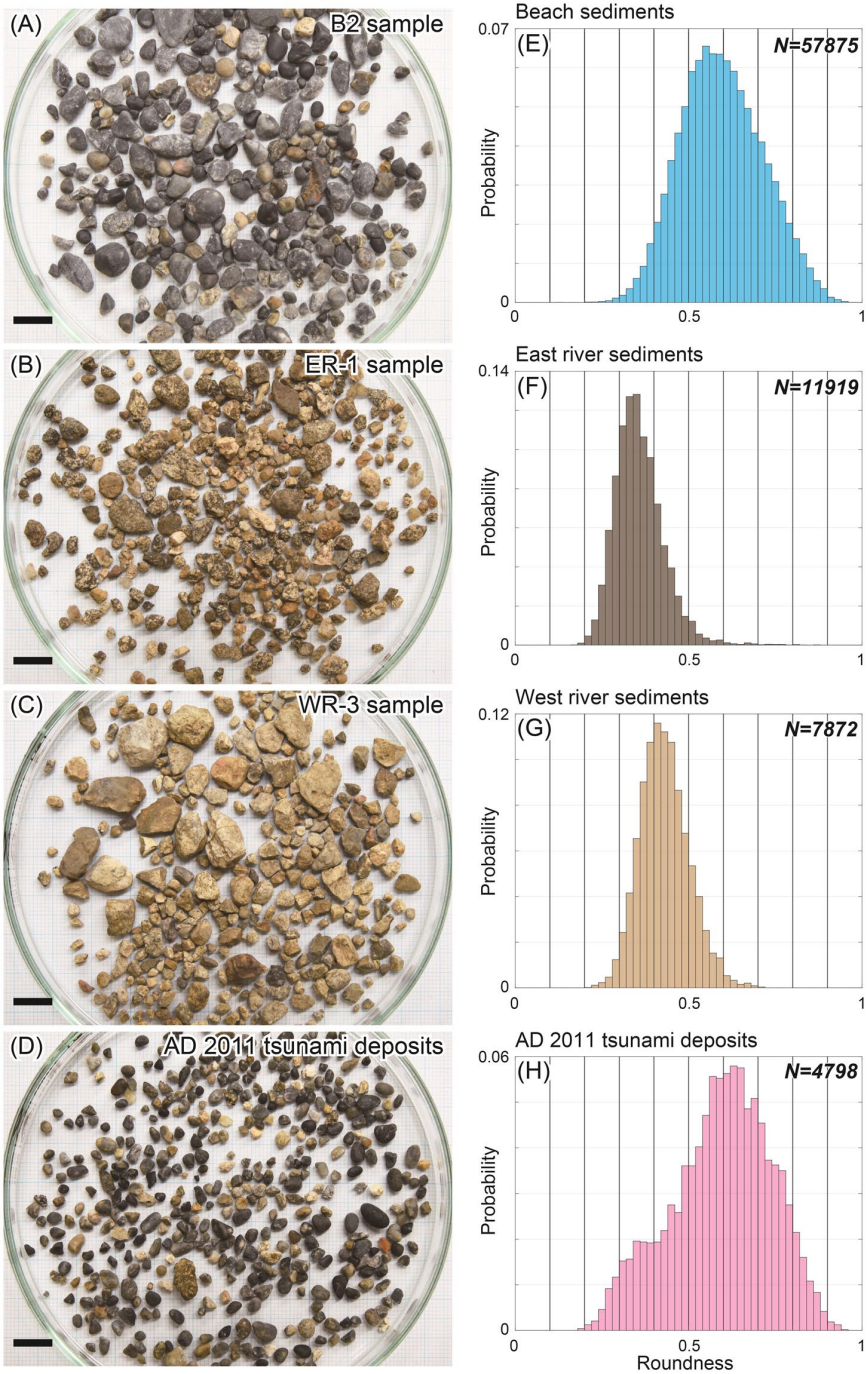
Photographs and roundness distributions of gravel particles in source materials and AD 2011 tsunami deposits (E1 tsunami deposits). (**A**–**D**) Photographs of samples. (**E**–**H**) The histograms show the roundness distributions of the beach, river and AD 2011 tsunami deposits (E1 tsunami deposits). To obtain the roundness distributions, we used only particles 2–8 mm in diameter. N = number of particles. The scale bars in the photographs are 1 cm.

We first measured the roundness values of gravel particles in beach sediments and in fluvial sediments at two locations (the east and west rivers) for comparison with tsunami deposits. On 19–20 July 2017, we sampled from two beach sites (B1 and B2) and six tributary sites (WR1–WR3 and ER1–ER3) where the AD 2011 Tohoku-oki tsunami did not inundate (Fig. 1B). The roundness values showed clear differences (Fig. 2E–G): the gravel particles of the beach sediments were rounded, reflecting abrasion by sea waves, and those of the fluvial sediments were angular. The roundness distribution of the E1 tsunami deposits (Fig. 2H) can be interpreted as a mixture of these source materials because there are at least two modes in the roundness distribution, and these modes correspond to the modes of fluvial and beach sediments (Fig. 2E–G). We therefore assumed that the studied tsunami deposits are composed of beach and fluvial materials and calculated the mixture ratios of these materials.

**Figure 3:**
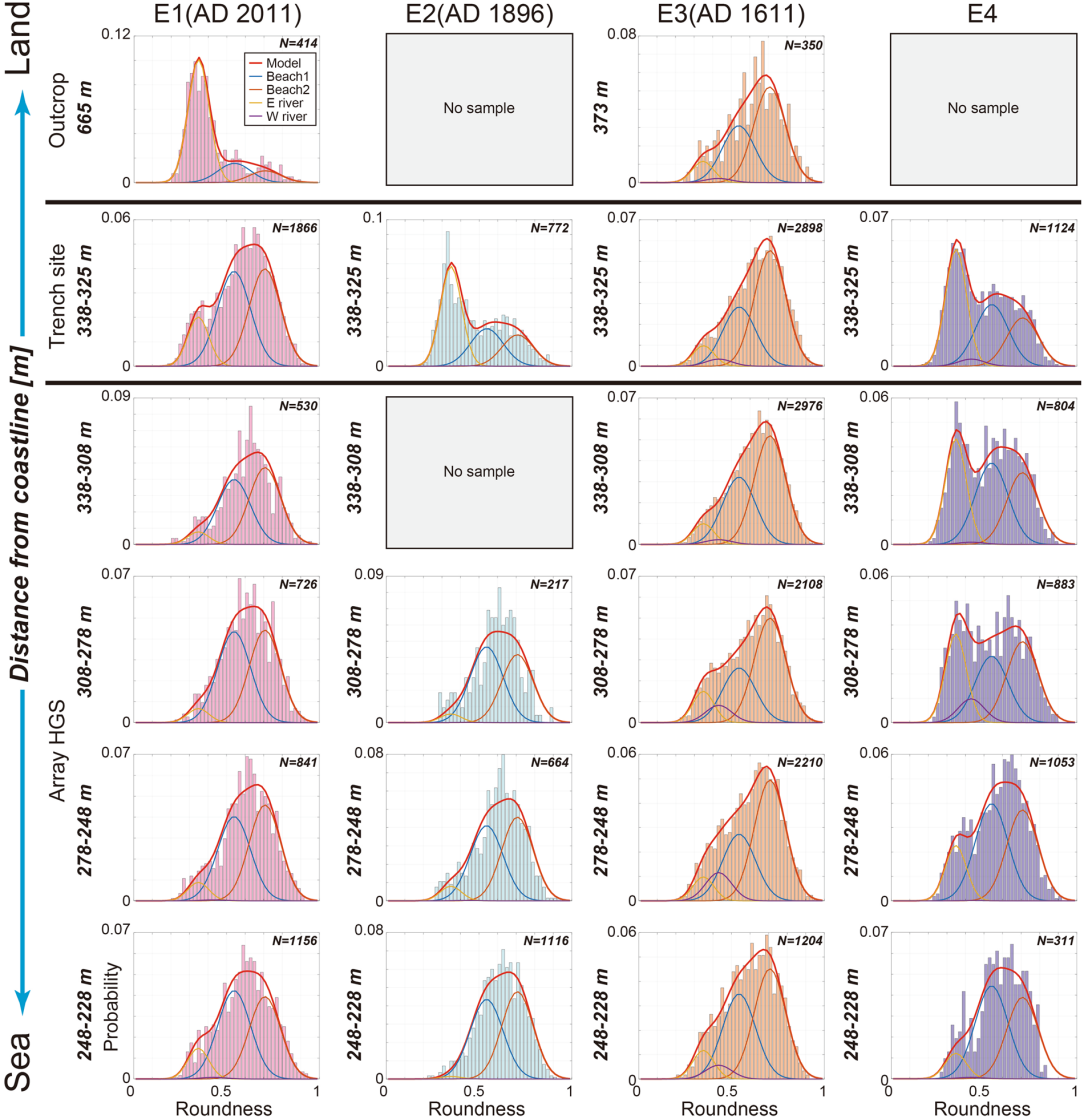
Changes in the roundness distribution of tsunami deposits E1 to E4 with distance from the coast. The numbers to the left of the histograms show the sample site distances from the coastline, in bins of 20 or 30 m (except at the top two rows). N = number of particles. The original data are provided in Supplementary Table S3.

We subsequently analysed 164 tsunami deposit samples taken from 56 sites, including the trench site, drilling sites and outcrop sites along a line orthogonal to the coast (Fig. 1B). In the E1–E3 tsunami deposits, for which the IDs are already known^19,25–27^, we found different trends of mixture ratio changes in the deposits from seaside to inland (Fig. 3). Gravels of the E2 tsunami deposits, which have a short ID, were associated with a high fluvial deposit content at the trench site 338 m inland. In contrast, gravels of the E1 and E3 tsunami deposits, which have long IDs (>1000 m), showed a low fluvial content at the trench site 338 m inland, but the fluvial content of the E1 tsunami deposits increased to more than 50% at a distance 665 m from the coastline.

We consider this increase in fluvially sourced gravels with inland distance to reflect tsunami transport processes. At the shore, run-up waves would mainly erode and transport beach sediments, resulting in high beach gravel contents in seaside tsunami deposits. In contrast, return waves would mainly erode and transport fluvial sediments (river, talus, and fan deposits), thereby resulting in high fluvial gravel contents in inland tsunami deposits. This relation indicates a correlation between changes in the mixture ratio away from the coast and tsunami sediment transport.

**Figure 4:**
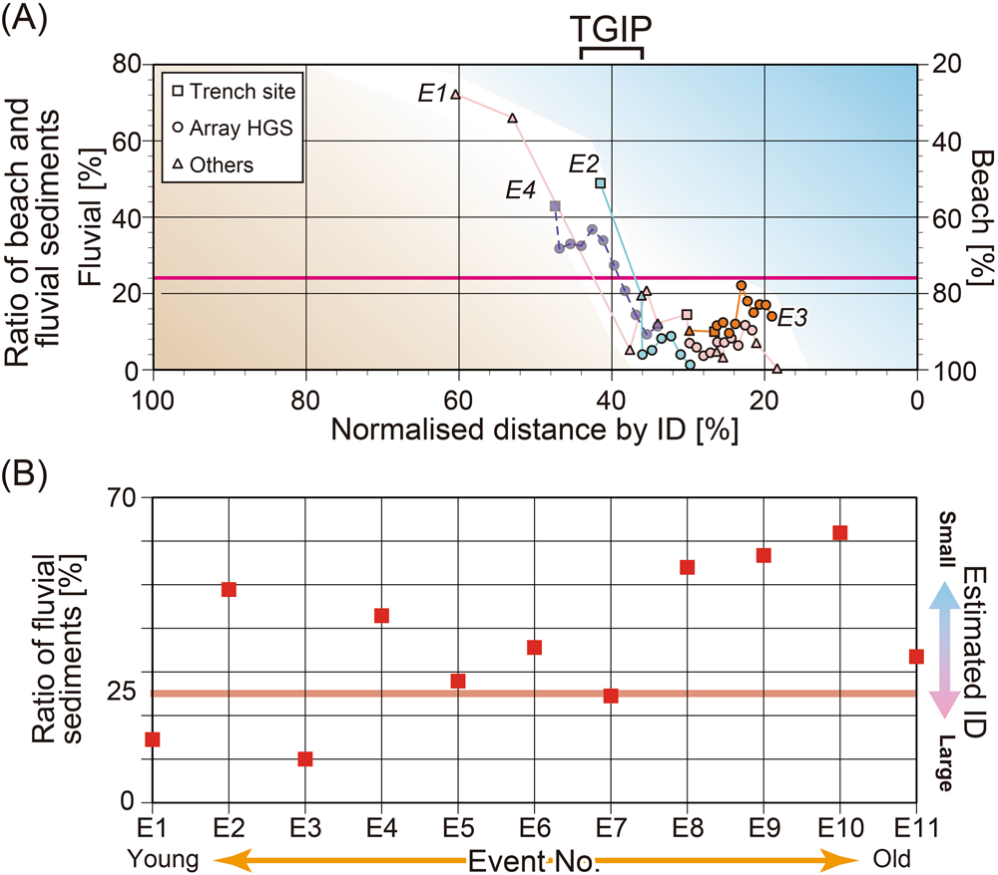
Ratios of fluvial sediments in tsunami deposits. (**A**) Relationship between the mixture ratios of beach and fluvial sources in tsunami deposits and normalised distance from the coastline. The distances of the E1 to E3 tsunami deposits from the coastline are normalised by their IDs, which are provided in Table 1. For the E4 tsunami deposit, we plotted data assuming that the ID was 675 m. (**B**) Mixture ratio of fluvial sediments in tsunami deposits (E1 to E11) at the trench site. Original data are provided in Supplementary Table S4. The right label indicates the estimated IDs assuming that the palaeo-coastline was in the same position as at present.

**Figure 5:**
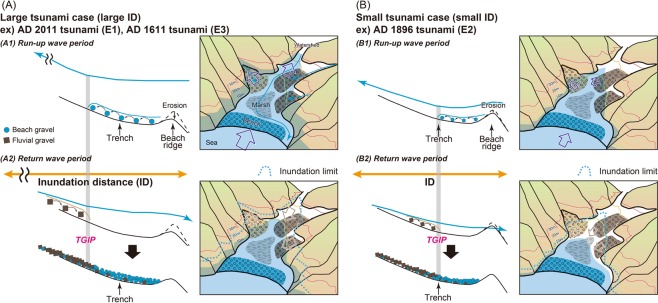
Schematic illustration showing the relationship between tsunami size and the transport of gravel particles. Schematic profiles showing the transport process of tsunami deposits for a large (**A**) and a small (**B**) tsunami. The arrows and arrow sizes in the bird’s eye view maps indicate the flow directions and relative flow speeds, respectively.

Distances of the E1–E3 tsunami deposit sample locations from the coast were normalised by each event’s ID (Fig. 4A), and abrupt changes in the mixture ratio were identified at the same percentile for the E1–E3 events. These steps mean that the distribution pattern of the composition of source materials in tsunami deposits from the coast to each inundation limit is consistent, regardless of the tsunami magnitudes. We call this point the tsunami gravel inflection point (TGIP), where the fluvial sediment ratio markedly changes from less than 25% to more than 40% (Fig. 4A). That is, the TGIP represents the balance point of sediment transport of gravels between the run-up and return waves of tsunamis. As illustrated schematically in Fig. 5, when the ID, which reflects the tsunami magnitude, changes, the TGIP moves landward or coastward in response.

### IDs of palaeo-tsunamis during the last 4000 years

Based on the above relationship, we could use the mixture ratios of palaeo-tsunami deposits obtained not only at multiple sites but also at one site to estimate the TGIPs associated with palaeo-tsunamis. First, using the distribution of mixture ratios of the E4 tsunami deposits (970–1290 cal yr BP^24^), we estimated the E4 TGIP and ID as approximately 280 m and 700 m from the coastline, respectively (Fig. 4A). Considering palaeo-topography and long-term crustal movement^22^, we assumed that the ca. 1000 cal yr BP palaeo-coastline was almost the same as the present coastline. Therefore, the estimated E4 ID (700 m) corresponds to a 10-m run-up height. We thus deduced that the palaeo-tsunami responsible for the E4 tsunami deposits was similar to or smaller than the AD 1896 Meiji Sanriku tsunami that is correlated with the E2 tsunami deposits. Next, we estimated the relative TGIP locations of the E5 to E11 tsunami deposits at the trench site (see Supplementary Fig. S2). The TGIPs of E5–E7 and E11 tsunami deposits were estimated to be near the trench site, whereas those of the E8–E10 tsunami deposits were deduced to be on the seaward side of the trench because their proportions of fluvial sediments significantly exceeded 25% (Fig. 4B). We could estimate the relative TGIP locations of E5 to E11 tsunami deposits for the trench site. However, precise palaeo-coastline restoration is required to estimate robust IDs and inundation heights.

The parameters (e.g., inundation height and distance) required to describe the actual tsunami size reflect not only tsunami magnitude but also various local factors, such as topography, air pressure, tides, and vegetation; i.e., these parameters are complex. Our new approach allows us to estimate ID directly using a spatially continuous roundness distribution. Moreover, data from only one site provide the opportunity to constrain the tsunami magnitude using the deduced TGIP position. By using the ID and TGIP estimated by our method, it is possible to estimate palaeo-tsunami run-up height and/or inundation area, allowing establishment or revision of tsunami generation models and tsunami risk assessments.

## Conclusions

The main finding of this study is that the mixture ratios of the source materials in tsunami deposits abruptly change at the same inundation distance percentile, regardless of the tsunami magnitude. This fact provides the novel possibility that inundation distance can be estimated directly from tsunami deposits at any other beach with sand and gravel, which occupy 31% of the ice-free coast on Earth^28^. That is, although the “tsunami gravel inflection point” would be variable by site due to local topography, this site-specific TGIP allows us to estimate an absolute palaeo-tsunami inundation distance from tsunami deposits. Moreover, we may be able to expand this idea to other parameters (e.g., heavy mineral composition, rock type content and terrestrial material content) to estimate tsunami magnitude from palaeo-tsunami deposits themselves. This new approach allows us to improve the understanding of palaeo-tsunami generation and will contribute to disaster prevention along coastal regions.

## Methods

We analysed the shapes of gravel particles more than 2 mm in diameter from tsunami deposits and modern sediments in beach and rivers. For tsunami deposits, we used the E1 to E11 tsunami deposits in the block samples obtained from the west, east, and south walls of the Koyadori trench^21^, Handy Geo-slicer^29,30^ (hereafter HGS) samples^31^ and outcrop samples (Fig. 1B). In the trench, the E1–E11 tsunami deposits were correlated because of their lateral continuity along the trench walls. In the HGS cores and outcrops, the E1–E4 tsunami deposits were also correlated with confidence because the HGS cores were drilled at 2.5 m intervals from the trench and the outcrop was exposed by construction of a shallow temporary canal. We also analysed modern samples obtained from two beach sites (B1 and B2) and six tributary sites (WR1–WR3 and ER1–ER3). On 19 July 2017, we sampled fluvial sediments on the riverbed at six sites (WR1–WR3 on the west tributary and ER1–ER3 on the east tributary; Fig. 1B and Supplementary Fig. S1C,D), where the AD 2011 Tohoku-oki tsunami did not inundate. On 20 July 2017, we sampled beach sediments on the surface at two beach survey lines (B1 and B2; Fig. 1B and Supplementary Fig. S1A,B). We sieved 2 mm larger particles (gravels) from washed samples. Then, we measured the roundness values of these gravels using the MATLAB program as shown below.

First, we arranged 200–400 gravel particles on a tray by hand such that they were not touching and took high-resolution photographs. Photographs were taken under front- and back-lit conditions using a NIKON D810 camera with AF-S Micro Nikkor 60 mm f/2.8 G ED and were recorded in RAW image format (see Supplementary Fig. S3). RAW files were processed into 328 to 343 pixels/cm JPEG images using Photoshop CS6.

Second, we analysed JPEG images taken under back-lit conditions in order to simplify the estimation of gravel boundaries using MATLAB (R2017b) and its toolboxes (Image Processing Toolbox, Statistics and Machine Learning Toolbox, Curve Fitting Toolbox, and Computer Vision System Toolbox). The JPEG true colour images were converted to grey scale intensity images (I) by forming a weighted sum of the R, G, and B components using a built-in function based on the following formula:

$${\rm{I}}=0.2989{\rm{R}}+0.5870{\rm{G}}+0.1140{\rm{B}}$$

After upsizing to 656 to 686 pixel/cm resolution using bicubic interpolation to avoid jagged edges on the particle boundaries, greyscale images were binarised using a locally adaptive threshold, specified as intensity values using the built-in function^32^.

Finally, we measured the roundness values of binarised gravel images using a particle roundness computation in the MATLAB program^33^ with three constrained parameters. This program first estimates smoothed boundaries calculated from measured pixel points using a built-in function based on locally weighted scatter plot smoothing (LOESS) specified by *α*, which is a smoothing span described as a percentage of the total number of data points. The program subsequently divides smoothing boundaries into line segments at key points and uniquely identifies corner points from these key points. The segmentation is specified by a threshold *δ*_0_, which is the maximum allowed divergence between particle curve and segments, indicating that the corner identification is critically controlled by this parameter. Finally, this program finds the best-fit circle for each corner using the corner points. The fitness is controlled by threshold T/R, where T and R are the minimum distance and radius of the fit circle, respectively. The appropriate tangent circle is found where T approximately equals R. Roundness is defined as the size ratio of the average size of corner circles to the maximum inscribed circle of the particle; therefore, measuring roundness is critically controlled by these three parameters.

Because these parameters are mainly affected by the image resolution, we need to decide the best parameters. The best-fit parameters were estimated by comparison with manually measured roundness data from the same photographs using a grid search method, resulting in α, δ0 and T/R values of 0.06, 0.017, and 0.996, respectively. Using these best parameters, we measured the roundness of source materials and palaeo-tsunami deposits and estimated their distribution.

To calculate the mixture ratio, assuming that the roundness of the three source materials follows a normal distribution, we calculated their means and standard deviations to explain their roundness distribution in the following regions: beach (B1 and B2), west river (WR1–WR3), and east river (ER1–ER3). However, it is difficult to fit the roundness distribution of beach sediments to a single normal distribution; thus, we set two normal distributions to explain the distribution. Finally, we set four endmember parameters (see Supplementary Table S2) representing the roundness distribution of source materials.

We used only 2–8 mm size gravels from the three source materials for calculation because >98% of gravel in all tsunami deposits ranges from 2–8 mm. We assumed that tsunami deposits are a simple mixture of materials from beach and fluvial sediments eroded by tsunamis because tsunami deposits hardly contain chipped stones. Therefore, the roundness distribution of tsunami deposits could be explained by a Gaussian mixture distribution. Finally, we calculated the best fit mixture ratios using the least squares method by changing the four endmember ratios.

## Supplementary information

Supplementary Figures and Tables
