# Supplementary material for: Palaeo-tsunami inundation distances deduced from roundness of gravel particles in tsunami deposits
Source: Sci Rep. 2019 Jul 16;9:10251. doi: 10.1038/s41598-019-46584-z (PMC6635351; doi:10.1038/s41598-019-46584-z)
Supplement: Supplementary file 1 — Supplementary Figures and Tables [file 41598_2019_46584_MOESM1_ESM.pdf]

Palaeo-tsunami inundation distances deduced from roundness of gravel particles in tsunami deposits

Daisuke Ishimura*[1], Keitaro Yamada *[2]

1. Department of Geography, Tokyo Metropolitan University

2. Research Centre for Palaeoclimatology, Ritsumeikan University

*: Both authors contributed equally to this work.
Correspondence and requests for materials should be addressed to D.I. (email: ishimura@tmu.ac.jp).

Supplementary Figures and Tables

Supplementary Table S1 Estimated ages of the E4–E11 tsunami deposits

Event No.	Estimated age (2σ) [cal yr BP]
E4	1290–970
E5	1710–1440
E6	2220–1690
E7	2710–2240
E8	2860–2580
E9	3240–2820
E10	3430–3210
E11	3880–3420

This is after reference No. 24. BP stands for "Before Present" and 0 BP is difined as AD 1950.

Supplementary Table S2 Endmember parameters used for mixture ratio calculations

Name	Mean	Standard deviation
Beach 1	0.5405	0.0885
Beach 2	0.7068	0.0846
East river	0.3477	0.0570
West river	0.4305	0.0703

Supplementary Table S3 Mixture ratio calculation results for Figure 3

Event No.	Sample name	Distance from coastline [m]	Beach[%]	W river[%]	E river[%]
E1	Trench	338–325	85.4	0.1	14.4
	HGS 0–30 m	338–308	94.4	0.1	5.5
	HGS 30–60m	308–278	95.2	0.0	4.8
	HGS 60–90m	278–248	93.3	0.4	6.3
	HGS 90–110m	248–228	88.7	0.7	10.5
E2	Trench	338–325	51.1	0.2	48.7
	HGS 30–60m	308–278	96.1	0.1	3.8
	HGS 60–90m	278–248	94.1	0.2	5.7
	HGS 90-110m	248–228	98.9	0.1	1.0
E3	Trench	338–325	90.0	3.0	7.0
	HGS 0–30 m	338–308	90.8	2.0	7.1
	HGS 30–60m	308–278	82.0	7.3	10.7
	HGS 60–90m	278–248	82.8	10.2	7.0
	HGS 90–110m	248–228	86.6	5.0	8.4
E4	Trench	338–325	57.2	3.0	39.9
	HGS 0–30 m	338–308	68.2	0.8	30.9
	HGS 30–60m	308–278	65.4	8.6	26.0
	HGS 60–90m	278–248	83.7	0.1	16.2
	HGS 90–110m	248–228	90.6	0.1	8.7

Supplementary Table S4 Mixture ratio calculation results for Figure 4

Event No.	Sample name	Distance from coastline [m]	Altitude [m]	Number of particles	Beach[%]	W river[%]	E river[%]
E1	HGS 0–20m	338–318	2.7–2.9	399	93.0	0.1	6.9
	HGS 10–30m	328–308	2.6–2.8	529	94.1	0.2	5.7
	HGS 20–40m	318–298	2.6–2.7	476	96.3	0.1	3.6
	HGS 30–50m	308–288	2.5–2.6	643	95.4	0.3	4.2
	HGS 40–60m	298–278	2.5–2.6	596	92.7	3.1	4.2
	HGS 50–70m	288–268	2.4–2.5	338	92.7	0.3	6.9
	HGS 60–80m	278–258	2.3–2.5	502	91.7	0.2	8.1
	HGS 70–90m	268–248	2.3–2.4	841	93.6	0.4	6.0
	HGS 80–100m	258–238	2.2–2.3	968	88.3	0.0	11.7
	HGS 90–110m	248–228	2.2–2.3	1156	89.6	1.5	8.9
	Loc. 1 (outcrop)	202	2.6	1213	99.5	0.2	0.2
	Loc. 2 (outcrop)	232	2.1	670	92.9	0.4	6.7
	Loc. 3 (outcrop)	280	2.5	378	96.7	0.9	2.4
	Loc. 4 (HGS)	289	2.5	276	95.2	0.0	4.8
	Loc. 6 (outcrop)	374	3.5	955	87.8	0.1	12.1
	Loc. 7 (HGS)	390	3.6	1068	79.2	0.0	20.8
	Loc. 8 (outcrop)	414	4.0	1408	94.7	0.4	4.9
	Loc. 9 (outcrop)	583	9.2	576	33.9	2.4	63.7
	Loc. 10 (outcrop)	665	11.8	414	27.7	0.4	71.9
E2	HGS 40–60m	298–278	2.5–2.6	216	96.0	0.6	3.4
	HGS 50–70m	288–268	2.4–2.5	381	95.0	0.1	5.0
	HGS 60–80m	278–258	2.3–2.5	324	91.8	0.1	8.1
	HGS 70–90m	268–248	2.3–2.4	501	91.2	0.5	8.3
	HGS 80–100m	258–238	2.2–2.3	1068	96.0	0.4	3.6
	HGS 90–110m	248–228	2.2–2.3	1116	98.8	0.1	1.2
	Loc. 4 (HGS)	289	2.5	466	80.4	0.3	19.2
E3	HGS 0–20m	338–318	2.7–2.9	2115	88.4	3.5	8.1
	HGS 10–30m	328–308	2.6–2.8	1832	87.6	5.0	7.4
	HGS 20–40m	318–298	2.6–2.7	1622	90.4	6.0	3.6
	HGS 30–50m	308–288	2.5–2.6	1222	87.9	4.0	8.0
	HGS 40–60m	298–278	2.5–2.6	1509	77.9	10.5	11.6
	HGS 50–70m	288–268	2.4–2.5	2138	81.9	9.7	8.3
	HGS 60–80m	278–258	2.3–2.5	1741	85.0	7.4	7.6
	HGS 70–90m	268–248	2.3–2.4	1494	82.9	9.7	7.4
	HGS 80–100m	258–238	2.2–2.3	1250	83.0	7.3	9.7
	HGS 90–110m	248–228	2.2–2.3	1204	86.0	6.0	8.0
	Loc. 5 (outcrop)	373	3.4	350	89.7	2.0	8.3
E4	HGS 0–20m	338–318	2.7–2.9	393	68.2	1.3	30.5
	HGS 10–30m	328–308	2.6–2.8	656	67.1	1.0	32.0
	HGS 20–40m	318–298	2.6–2.7	505	67.5	0.8	31.7
	HGS 30–50m	308–288	2.5–2.6	498	63.2	2.1	34.7
	HGS 40–60m	298–278	2.5–2.6	730	66.1	10.5	23.4
	HGS 50–70m	288–268	2.4–2.5	1034	72.6	7.0	20.4
	HGS 60–80m	278–258	2.3–2.5	629	79.2	0.4	20.3
	HGS 70–90m	268–248	2.3–2.4	923	85.6	0.1	14.3
	HGS 80–100m	258–238	2.2–2.3	645	90.8	0.4	8.9
	HGS 90–110m	248–228	2.2–2.3	311	88.6	0.4	11.0
E5	Trench	338–325	2.8–3.0	1371	72.1	6.1	21.8
E6	Trench	338–325	2.8–3.0	2823	64.3	10.1	25.5
E7	Trench	338–325	2.8–3.0	1209	75.5	2.2	22.3
E8	Trench	338–325	2.8–3.0	398	46.1	23.9	30.1
E9	Trench	338–325	2.8–3.0	879	43.2	21.3	35.4
E10	Trench	338–325	2.8–3.0	510	38.0	13.6	48.3
E11	Trench	338–325	2.8–3.0	506	66.5	19.7	13.8

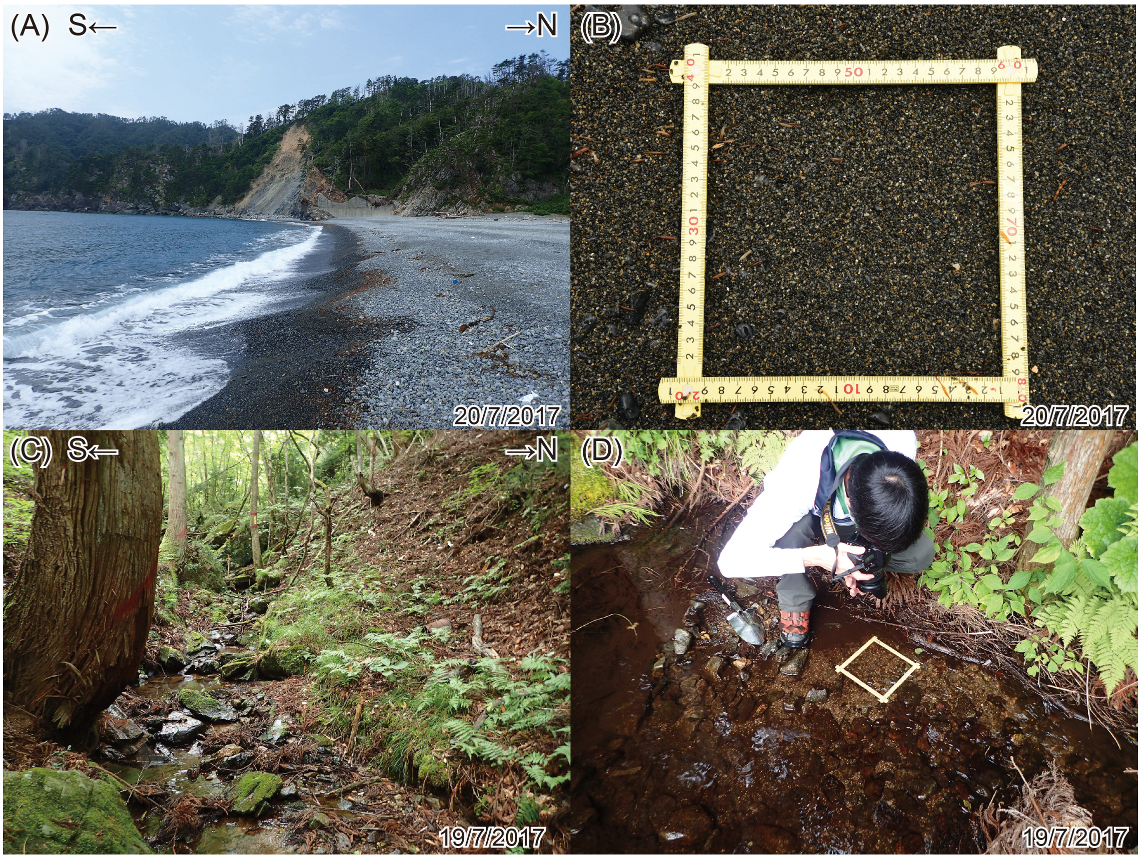

Supplementary Fig. S1 Landscapes at the sampling sites

(A) Beach in 2017. (B) Close-up photograph of beach sediments. (C) West river in 2017.

(D) East river in 2017.

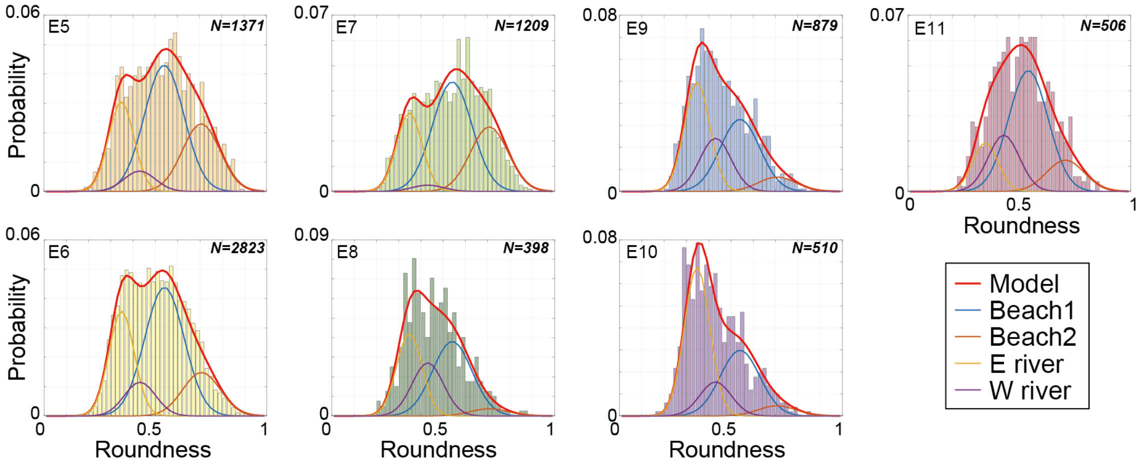

Supplementary Fig. S2 Roundness distributions of the E5 to E11 tsunami deposits at the trench site

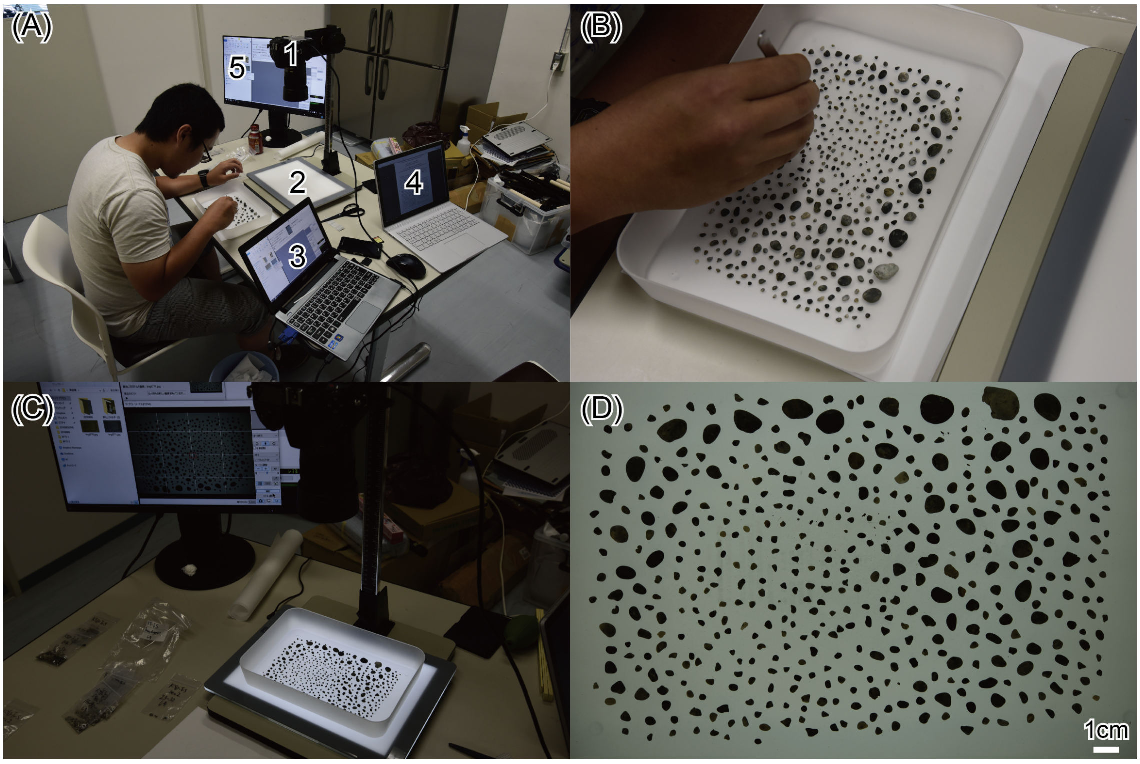

Supplementary Fig. S3 Photographic procedure

(A) Photographic system. 1: Camera (the camera body and lens are a Nikon D810 and a Nikon AF-S Micro NIKKOR 60 mm f/2.8G ED, respectively). 2: Tracing table. 3: Computer for photography. 4: Computer for image analysis. 5: Monitor for sample positioning. (B) Placement of gravel particles by hand. (C) Photography of gravel particles. (D) Example photograph of gravel particles. The image resolution is 7360 × 4912 pixels, and the image scale is 328 to 343 pixels per centimetre.
